# Predicting the Effectiveness of a Mindfulness Virtual Community Intervention for University Students: Machine Learning Model

**DOI:** 10.2196/50982

**Published:** 2024-05-13

**Authors:** Christo El Morr, Farideh Tavangar, Farah Ahmad, Paul Ritvo, MVC Team

**Affiliations:** 1 School of Health Policy and Management York University Toronto, ON Canada; 2 Lawrence S. Bloomberg Faculty of Nursing University of Toronto Toronto, ON Canada; 3 Kinesiology & Health Science York University Toronto, ON Canada; 4 See Acknowledgments

**Keywords:** machine learning, virtual community, virtual care, mindfulness, depression, anxiety, stress, students, online, randomized controlled trial, Canada, virtual, artificial intelligence, symptoms, behavioral therapy, sociodemographic, mindfulness video, online video

## Abstract

**Background:**

Students’ mental health crisis was recognized before the COVID-19 pandemic. Mindfulness virtual community (MVC), an 8-week web-based mindfulness and cognitive behavioral therapy program, has proven to be an effective web-based program to reduce symptoms of depression, anxiety, and stress. Predicting the success of MVC before a student enrolls in the program is essential to advise students accordingly.

**Objective:**

The objectives of this study were to investigate (1) whether we can predict MVC’s effectiveness using sociodemographic and self-reported features and (2) whether exposure to mindfulness videos is highly predictive of the intervention’s success.

**Methods:**

Machine learning models were developed to predict MVC’s effectiveness, defined as success in reducing symptoms of depression, anxiety, and stress as measured using the Patient Health Questionnaire-9 (PHQ-9), the Beck Anxiety Inventory (BAI), and the Perceived Stress Scale (PSS), to at least the minimal clinically important difference. A data set representing a sample of undergraduate students (N=209) who took the MVC intervention between fall 2017 and fall 2018 was used for this secondary analysis. Random forest was used to measure the features’ importance.

**Results:**

Gradient boosting achieved the best performance both in terms of area under the curve (AUC) and accuracy for predicting PHQ-9 (AUC=0.85 and accuracy=0.83) and PSS (AUC=1 and accuracy=1), and random forest had the best performance for predicting BAI (AUC=0.93 and accuracy=0.93). Exposure to online mindfulness videos was the most important predictor for the intervention’s effectiveness for PHQ-9, BAI, and PSS, followed by the number of working hours per week.

**Conclusions:**

The performance of the models to predict MVC intervention effectiveness for depression, anxiety, and stress is high. These models might be helpful for professionals to advise students early enough on taking the intervention or choosing other alternatives. The students’ exposure to online mindfulness videos is the most important predictor for the effectiveness of the MVC intervention.

**Trial Registration:**

ISRCTN Registry ISRCTN12249616; https://www.isrctn.com/ISRCTN12249616

## Introduction

Students’ mental health crises were recognized before the COVID-19 pandemic and deepened during the pandemic. University students are experiencing an increase in psychological distress on North American campuses. A student survey of 32 Canadian postsecondary institutions reported high anxiety (56.5%), hopelessness (54%), seriously depressed mood (37.5%), and overwhelming anger (42%) [1]. A similar survey in 2016 revealed higher distress levels [2]. In 2013, a study of 997 students at York University (site of this study) indicated that 57% reported depression scores sufficient for diagnosable clinical depression, while 33% reported anxiety scores in ranges typically indicative of panic disorder and generalized anxiety disorder [3]. The situation appears similar at universities in the United States [4,5] and worldwide; in 2018, the World Health Organization reported increasing mental disorders in college and university students worldwide [6]. Mental health challenges among university students demand attention. This is a vulnerable period, as 70% of mental health problems emerge before the age of 25 years. Without intervention, these problems can worsen and hinder students’ personal and academic success [7]. COVID-19 has negatively impacted university students’ mental health [8-10].

University student distress is both an individual and societal challenge. Losses in productivity during the study and at work due to distress and mental disorders are associated with indirect but significant economic burdens [11]. Canadian estimates show that mental disorders cost nearly US $37 billion yearly, with 9.8% due to direct medical costs, 16.6% and 18.2% due to long-term loss and short-term work loss, respectively, and 55.4% due to the loss of healthy function (ie, loss of the utilities of vision, hearing, speech, mobility, dexterity, emotion, cognition, and pain as assessed in the Health Utilities Index Mark 3 system) [12].

While mental distress and disorder are becoming more prevalent in students, the counseling offered in colleges and universities needs to catch up with demand. For example, from 2007 to 2012, full-time enrollment in the Ontario college system increased from 167,000 to 210,600 (a 26% increase), while the number of counselors employed in the college system increased from 146 to 152.7 (a 4.6% increase) [13]. This discrepancy leaves students underserved and counselors overwhelmed amid the increasing distress [14].

Mindfulness-based interventions have been demonstrated to positively impact psychological and physical health [15-17], with multiple meta-analyses demonstrating positive impacts in clinical and nonclinical populations [18-22]. However, with large numbers of students (50,000 to 60,000 on some campuses), there may not be enough trained personnel to convey helpful mindfulness-based practices directly. Instead, in the eHealth domain, virtual communities (VCs) [23], that is, online communities, have been used in health care to provide e-education tools and online support to empower active participants in health enhancement [24-26]. VCs can scale up mindfulness interventions at lower costs to a broader range of students, especially those restricted from attending clinics due to time-place discontinuities. VCs preserve anonymity (with reduced stigmatization) while promoting voluntary, supportive, interpersonal connections.

We developed a web-delivered mindfulness program (mindfulness virtual community [MVC]) to reduce symptoms of depression, anxiety, and stress in university students and conducted a randomized controlled trial (RCT) targeting university students at a Canadian university to examine its effectiveness. Following a successful RCT [26-29], we wanted in this secondary analysis (1) to develop a machine learning (ML) model to predict the effectiveness of the online mindfulness intervention on mental health outcomes using sociodemographic and self-reported features and (2) to investigate if exposure to mindfulness videos was highly predictive of the intervention’s success.

## Methods

### Prediction Problem

This study aims to predict the effectiveness (ie, success vs nonsuccess) of the online mindfulness intervention on mental health outcomes; as such, this is a retrospective prognostic analysis of a classification problem per individual (ie, participants in the MVC mindfulness intervention).

### Data Set Source

**Figure 1:**
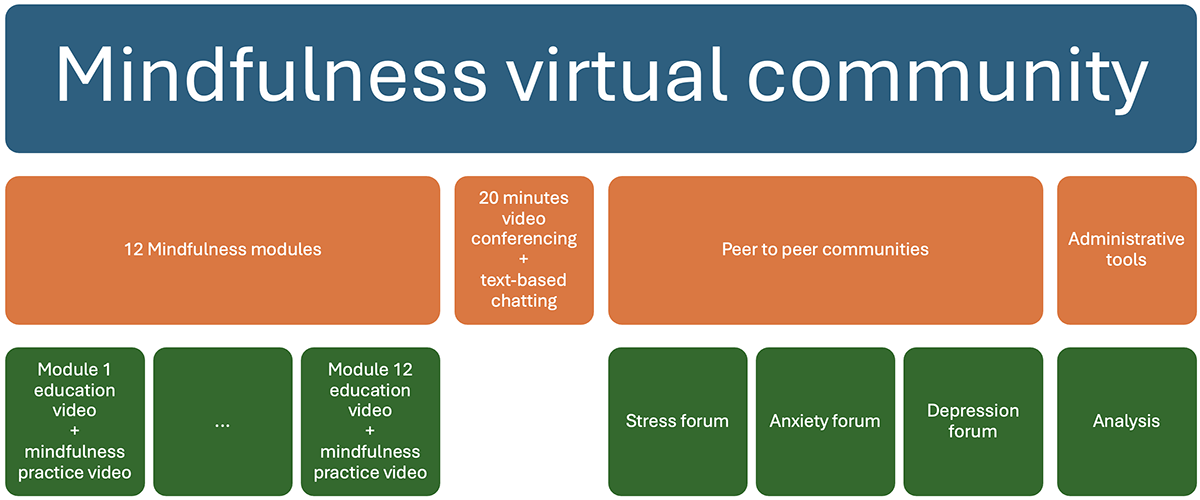
The mindfulness virtual community design.

This is a retrospective analysis, where we analyzed an anonymized data set. The data were deidentified, and consent was obtained during the RCT; no further consent was sought for this secondary data analysis since nonidentifiable data were used. The data set was collected via an RCT described in detail elsewhere [28]. The parent study design consisted of a 2-arm parallel-design RCT, comparing a group assigned to the web-based MVC program to a waitlist control group. Participants in the study were students who were at least 18 years of age, reported English language fluency, self-reported high confidence in completing the study, and actively enrolled in an undergraduate program. This paper is based on the MVC intervention sample recruited in fall 2017, winter 2017, and fall 2018. The MVC intervention was an 8-week program and was comprised of three components: (1) 12 online videos for mental health education; (2) 3 anonymous discussion boards on depression, anxiety, and stress; and (3) anonymous, 20-minute group-based live videoconferences led by a mental health professional with training in mindfulness during which students could raise questions related to mindfulness (Figure 1).

Each of the 12 mental health modules consisted of 1 educational content video and 1 mindfulness practice video recorded in both male and female voices and offered in both high and low resolution (a total of 8 videos per module); participants could choose the type of video they wanted to watch for each module. The videos were available for participants 24 hours a day to watch or listen to on computers, phones, or tablets at their convenience. The module scripts and audio recordings were created by one of the investigators with extensive experience as a clinical psychologist and researcher in mindfulness. They were based on mindfulness and cognitive behavioral therapy principles and informed by the prior student-based focus group study [30,31]—the choice of moving and still images used in creating the videos involved collaborative work. The topics of the 12 modules included the following: overcoming stress, anxiety, and depression; mindfulness and being a student; mindfulness for better sleep; thriving in a fast-changing world; healthy intimacy; destigmatization; no more procrastination; pain reduction and mindfulness; healthy body image; healthier eating; overcoming trauma; and relationships with family and friends.

The primary RCT outcomes were depression, anxiety, and perceived stress, following hypotheses that symptom scores for depression, anxiety, and stress at T2 (after 8 weeks) would be significantly better in the MVC group when compared with the waitlist control group. The outcomes were measured with the following validated scales: Patient Health Questionnaire-9 (PHQ-9) [32], Beck Anxiety Inventory (BAI) [33], and Perceived Stress Scale (PSS) [34]. The secondary aim was to assess the impact of 3 elements of the MVC intervention on the outcomes. Participants also completed a sociodemographic questionnaire section at the T1 (baseline) survey.

### Ethical Considerations

The previous study received ethics approval from the Human Participant Research Committee (certificate e2016-345) of the York University. This ML study received ethics approval from the same committee (certificate e2023-012); the approval covers secondary analysis without additional consent. Participants in the original study had the option to receive an honorarium of CAD $50 (US $37.5) or 2% in course grade (for professors who gave this permission) or 3 credits (equivalent to 2% course grade) in the Undergraduate Research Participation Pool of the Department of Psychology. The participants’ data were anonymized.

### Participants

We aimed to build a model to predict who will likely benefit from the intervention, unlike the RCT study, where overall intervention effectiveness was determined (and supported by analysis) by comparing intervention and control groups. That is why we have analyzed intervention group data only to understand individual differences in response to the intervention.

### Data Preparation

The data set consisted of 209 students who took the MVC intervention during fall 2017, winter 2018, and fall 2018. The effectiveness of the intervention was determined using the minimal clinically important difference (MCID), that is, the level of reduction in symptoms that psychologists consider clinically meaningful, for each of the mental health outcomes. We adopted evidence from psychology that determines the MCID to be a 5-point reduction in PHQ-9 for depression [35,36], an 8.8-point reduction in BAI for anxiety [30,31], and an 11-point reduction in PSS for stress [37,38]. Any reduction equal to or above the MCID was labeled an effective intervention (label=1); otherwise, it was deemed ineffective (label=0).

To build a good prediction model from the training set, the data must be balanced. The class labels of the target variables, PHQ-9, BAI, and PSS, used in this study were not balanced. In our case, the percentage of instances with label=1 was extremely low: 50 (23%) for PHQ-9, 48 (24%) for BAI, and 8 (3.8%) for PSS, leading to a substantial imbalance. To alleviate the imbalanced data, we applied an oversampling method using the sklearn.resample function available in Python (version 3; Python Software Foundation).

### Missing Data

Missing data in the outcomes were 12 (5.7%) for BAI and PHQ-9 and 13 (6.2%) for PSS of the 209 records. Missing data for the outcomes were dropped from the data set. There were no missing values in the predictors.

### Labels and Features

The outcome variables were the 3 MCIDs associated with PHQ-9, BAI, and PSS being met or not for each instance. To investigate whether we can predict MVC’s effectiveness using sociodemographic and self-reported features, the following features were used: sex (male and female), country of birth (Canada and other), first language (English and other), education level (bachelor degree and other), ethnicity (White and non-White), marital status (married and other), age, number of weekly working hours, and self-rated health (poor, fair, good, very good, and excellent). To investigate the importance of exposure to mindfulness videos, in comparison with these features, in the prediction of intervention success, we added the total number of mindfulness videos watched to the previous data set.

### Algorithms

Seven different classification algorithms, representing different learning paradigms, were used in this study: logistic regression (LR), support vector machine (SVM), random forest (RF), decision tree (DT), k-nearest neighbor (KNN), adaptive boosting (AdaBoost), and gradient boosting that showed good performance in previous studies that targeted depression, anxiety, and stress [35,39,40]. The implementations of the classification algorithms provided in the scikit-learn ML library [41] were used. The data set was split into 80% for training and 20% for testing. Hyperparameter tuning for each algorithm was performed using a grid search over a 10-fold cross-validation on the training data set. The optimal hyperparameters for the classification algorithms and their values for the data set without exposure to videos and the data set with exposure to videos are presented in Tables 1 and 2, respectively.

Each classifier’s performance was compared with the best overall performance, leading to the selection of the best prediction model for the psychological outcomes. The classifiers’ performances were assessed based on several evaluation metrics, including the percentage of correctly classified instances or the accuracy, sensitivity, specificity, and area under the curve (AUC) of the receiver operating characteristic curve. The best performance, as measured by the AUC score, was chosen for each algorithm.

To evaluate the features’ importance in predicting intervention success, the data set with the total exposure to mindfulness videos was used to build predictive models. The RF algorithm was used to measure the features’ importance. The hyperparameters used for the classification algorithms and their values that provided the optimal model are presented in Table 2.

**Table 1:** Algorithms and their corresponding optimal hyperparameters found by grid search (data set without videos).

Algorithm		Parameters
**Logistic regression**		
	PHQ-9^a^	C=1, penalty=l1, solver=liblinear
	BAI^b^	C=1, penalty=l1, solver=liblinear
	PSS^c^	C=1, penalty=l1, solver=liblinear
**Support vector machine**		
	PHQ-9	C=10, γ=0.1, kernel=rbf
	BAI	C=10, γ=0.1, kernel=rbf
	PSS	C=10, γ=0.1, kernel=rbf
**Random forest**		
	PHQ-9	Max_features=auto, n_estimators=500, max_depth=8, criterion=entropy
	BAI	Max_features=auto, n_estimators=500, max_depth=8, criterion=gini
	PSS	Max_features=auto, n_estimators=500, max_depth=8, criterion=gini
**Decision tree**		
	PHQ-9	Max_leaf_nodes=59, random_state=42, min_samples_split=2, criterion=entropy
	BAI	Max_leaf_nodes=56, random_state=42, min_samples_split=2, criterion=entropy
	PSS	Max_leaf_nodes=16, random_state=42, min_samples_split=2, criterion=entropy
**K-nearest neighbor**		
	PHQ-9	N_neighbors=2, weight=distance, leaf size=27, P=1
	BAI	N_neighbors=2, weight=distance, leaf size=1, P=1
	PSS	N_neighbors=1, weight=dniform, leaf size=1, P=1
**Adaptive boosting**		
	PHQ-9	n-estimators=5000, max_depth=3, learning rate=0.5
	BAI	n-estimators=5000, max_depth=3, learning rate=0.9
	PSS	n-estimators=500, max_depth=3, learning rate=0.9
**Gradient boosting**		
	PHQ-9	Learning rate=0.05, max depth=6, n-estimators=100, subsample=0.9, max_features=none, min_samples_split=2
	BAI	Learning rate=0.02, max depth=10, n-estimators=1000, subsample=1.0, max_features=none, min_samples_split=2
	PSS	Learning rate=0.01, max depth=6, n-estimators=1000, subsample=0.9, max_features=sqrt, min_samples_split=2

^a^PHQ-9: Patient Health Questionnaire-9.

^b^BAI: Beck Anxiety Inventory.

^c^PSS: Perceived Stress Scale.

**Table 2:** Algorithms and their corresponding optimal hyperparameters found by grid search (data set with exposure to videos).

Algorithm		Parameters
**Logistic regression**		
	PHQ-9^a^	C=1, penalty=l1, solver=liblinear
	BAI^b^	C=0.1, penalty=l2, solver=newton-cg
	PSS^c^	C=100, penalty=l2, solver=lbfgs
**Support vector machine**		
	PHQ-9	C=10, γ=0.01, kernel=rbf
	BAI	C=1, γ=1, kernel=rbf
	PSS	C=1, γ=1, kernel=rbf
**Random forest**		
	PHQ-9	Max_features=auto, n_estimators=500, max_depth=7, criterion=entropy
	BAI	Max_features=auto, n_estimators=200, max_depth=8, criterion=gini
	PSS	Max_features=auto, n_estimators=500, max_depth=8, criterion=gini
**Decision tree**		
	PHQ-9	Max_leaf_nodes=39, random_state=42, min_samples_split=2, criterion=entropy
	BAI	Max_leaf_nodes=53, random_state=42, min_samples_split=3, criterion=gini
	PSS	Max_leaf_nodes=16, random_state=42, min_samples_split=2, criterion=gini
**K-nearest neighbor**		
	PHQ-9	N_neighbors=1, weight=uniform, leaf size=14, P=1
	BAI	N_neighbors=2, weight=distance, leaf size=1, P=1
	PSS	N_neighbors=2, weight=uniform, leaf size=1, P=1
**Adaptive boosting**		
	PHQ-9	n-estimators=500, max_depth=3, learning rate=0.5
	BAI	n-estimators=500, max_depth=3, learning rate=0.7
	PSS	n-estimators=2000, max_depth=3, learning rate=0.7
**Gradient boosting**		
	PHQ-9	Learning rate=0.5, max depth=50, n-estimators=50, subsample=0.9, max_features=sqrt, min_samples_split=2
	BAI	Learning rate=0.04, max depth=10, n-estimators=1000, subsample=0.5, max_features=none, min_samples_split=2
	PSS	Learning rate=0.03, max depth=8, n-estimators=1000, subsample=0.5, max_features=none, min_samples_split=2

^a^PHQ-9: Patient Health Questionnaire-9.

^b^BAI: Beck Anxiety Inventory.

^c^PSS: Perceived Stress Scale.

## Results

### Demographics

Table 3 presents the demographic characteristics of participants at baseline. Of 209 students, 73.2% (n=153) were female, 8.1% (n=17) were married, and 21.1% (n=44) were White. Most participants were born in Canada, and English was their first language. The median (IQR) of age, work hours per week, and the total number of mindfulness videos watched were 21 (19-23) years, 10 (0-18), and 16 (9-30), respectively.

**Table 3:** Characteristics of participants at baseline (N=209).

Characteristics		Values
**Sex, n (%)**		
	Male	56 (26.8)
	Female	153 (73.2)
**Marital status, n (%)**		
	Married	17 (8.1)
	Other	192 (91.9)
**Ethnicity, n (%)**		
	White	44 (21.1)
	Non-White	165 (78.9)
**Language, n (%)**		
	English	136 (65.1)
	Other	73 (34.9)
**Country of birth, n (%)**		
	Canada	119 (56.9)
	Other	90 (43.1)
**Education, n (%)**		
	High school diploma or General Education Development or college degree or certificate program	182 (87.1)
	Bachelor degree	27 (12.9)
**Self-reported general health, n (%)**		
	Poor or fair	43 (20.6)
	Good or very good or excellent	166 (79.4)
Age (years), median (IQR)		21 (19-23)
Average number of hours at work per week, median (IQR)		10 (0-18)
Total number of mindfulness videos watched, median (IQR)		16 (9-30)

### Objective 1: Predicting MVC’s Effectiveness Using Sociodemographic and Self-Reported Features

Table 4 summarizes the evaluated models’ performances: sensitivity, specificity, accuracy, and AUC, using 10-fold cross-validation.

The results showed that both gradient boosting (AUC=0.85 and accuracy=0.83) and DT (AUC=0.84 and accuracy=0.81) are slightly better compared to AdaBoost and KNN (AUC=0.82 and accuracy=0.80) as well as SVM (AUC=0.81 and accuracy=0.80) and outperformed the remaining classification algorithms for predicting a clinically significant reduction in PHQ-9. The best classifiers for predicting a clinically significant reduction in BAI were RF (AUC=0.93 and accuracy=0.93), followed by AdaBoost (AUC=0.92 and accuracy=0.92) and gradient boosting (AUC=0.87 and accuracy=0.87), which outperformed the remaining classifiers. Two classifiers, gradient boosting and DT, gained the perfect accuracy and AUC (AUC=1 and accuracy=1) for predicting a clinically significant reduction in PSS, followed by the near-perfect scores for SVM and AdaBoost (AUC=0.99 and accuracy=0.99). Meanwhile, LR had the lowest performance for PHQ-9, BAI, and PSS in terms of AUC (0.64, 0.75, and 0.73, respectively) and accuracy (0.66, 0.75, and 0.73, respectively).

The results were close to those found in the models built without video exposure. Gradient boosting (AUC=0.89 and accuracy=0.88) was the best predictor for a significant reduction in PHQ-9, followed closely by AdaBoost and DT (AUC=0.84 and accuracy=0.81), which outperformed the remaining classification algorithms. The best classifiers for predicting a clinically significant reduction in BAI were AdaBoost and SVM (AUC=0.93 and accuracy=0.93), followed closely by gradient boosting (AUC=0.91 and accuracy=0.92) and RF (AUC=0.90 and accuracy=0.90), which outperformed the remaining classifiers. Four classifiers, gradient boosting, AdaBoost, RF, and SVM, gained the perfect AUC and accuracy (AUC=1 and accuracy=1) for predicting a clinically significant reduction in PSS, followed by the near-perfect score for KNN (AUC=0.99 and accuracy=0.99) and DT (AUC=0.97 and accuracy=0.97). Meanwhile, LR had the lowest performance for PHQ-9, BAI, and PSS in terms of AUC (0.62, 0.60, and 0.79, respectively) and accuracy (0.63, 0.60, and 0.80, respectively).

Using the second data set (ie, enriched with the exposure to videos), RF was used to detect features’ importance in relation to the 3 outcomes. The most predictive feature for the PHQ-9, BAI, and PSS was the total exposure to the mindfulness videos, followed by the average number of working hours per week and age for PHQ-9 and BAI. In contrast, age and the average number of working hours per week were the second and third most important predictors for PSS, respectively.

**Table 4:** Classification report of the machine learning algorithms for outcomes.

Algorithm		AUC^a^	Accuracy	Sensitivity	Specificity
**Logistic regression**					
	PHQ-9^b^	0.64	0.66	0.57	0.72
	BAI^c^	0.75	0.75	0.75	0.75
	PSS^d^	0.73	0.73	0.73	0.74
**Support vector machine**					
	PHQ-9	0.81	0.80	0.90	0.75
	BAI	0.77	0.77	0.79	0.75
	PSS	0.96	0.96	1.0	0.91
**Random forest**					
	PHQ-9	0.78	0.76	0.87	0.69
	BAI	0.93	0.93	0.86	1.0
	PSS	0.99	0.99	1.0	0.97
**Decision tree**					
	PHQ-9	0.84	0.81	0.96	0.72
	BAI	0.84	0.83	0.93	0.75
	PSS	1.0	1.0	1.0	1.0
**K-nearest neighbor**					
	PHQ-9	0.82	0.80	0.91	0.72
	BAI	0.78	0.78	0.68	0.88
	PSS	0.96	0.96	1.0	0.91
**Adaptive boosting**					
	PHQ-9	0.82	0.80	0.91	0.72
	BAI	0.92	0.92	0.89	0.94
	PSS	0.99	0.99	1.0	0.97
**Gradient boosting**					
	PHQ-9	0.85	0.83	0.91	0.78
	BAI	0.87	0.87	0.86	0.88
	PSS	1.0	1.0	1.0	1.0

^a^AUC: area under the curve.

^b^PHQ-9: Patient Health Questionnaire-9.

^c^BAI: Beck Anxiety Inventory.

^d^PSS: Perceived Stress Scale.

### Objective 2: Importance of Exposure to Mindfulness Videos in Comparison With Sociodemographics and Self-Reported Features in Predicting Intervention Success

After the introduction of the total exposure to the mindfulness videos to the data set, new predictive models were built (Table 5).

**Table 5:** Classification report of the machine learning algorithms for outcomes (data set with exposure to videos).

Algorithm		AUC^a^	Accuracy	Sensitivity	Specificity
**Logistic regression**					
	PHQ-9^b^	0.62	0.63	0.57	0.67
	BAI^c^	0.60	0.60	0.61	0.59
	PSS^d^	0.79	0.80	0.85	0.74
**Support vector machine**					
	PHQ-9	0.78	0.75	0.96	0.61
	BAI	0.93	0.93	0.86	1.00
	PSS	1.00	1.00	1.00	1.00
**Random forest**					
	PHQ-9	0.84	0.83	0.87	0.81
	BAI	0.90	0.90	0.86	0.94
	PSS	1.00	1.00	1.00	1.00
**Decision tree**					
	PHQ-9	0.84	0.81	0.96	0.72
	BAI	0.80	0.80	0.86	0.75
	PSS	0.97	0.97	1.00	0.94
**K-nearest neighbor**					
	PHQ-9	0.81	0.78	0.96	0.67
	BAI	0.84	0.83	0.89	0.78
	PSS	0.99	0.99	1.00	0.97
**Adaptive boosting**					
	PHQ-9	0.84	0.81	0.96	0.72
	BAI	0.93	0.93	0.89	0.97
	PSS	1.00	1.00	1.00	1.00
**Gradient boosting**					
	PHQ-9	0.89	0.88	0.96	0.83
	BAI	0.91	0.92	0.86	0.97
	PSS	1.00	1.00	1.00	1.00

^a^AUC: area under the curve.

^b^PHQ-9: Patient Health Questionnaire-9.

^c^BAI: Beck Anxiety Inventory.

^d^PSS: Perceived Stress Scale.

## Discussion

### Principal Results

The study investigated the predictability of the effectiveness of an MVC designed for undergraduate students to reduce symptoms of depression, anxiety, and stress as measured by PHQ-9, BAI, and PSS. The effectiveness was measured by the MCID for PHQ-9, BAI, and SPSS. Several algorithms were used to predict the MCID.

### Predicting Intervention Success With Sociodemographic and Self-Reported Measures

We successfully built ML-based models that predicted the effectiveness of the MVC intervention. The highest AUC was achieved for gradient boosting to predict the intervention effectiveness for PHQ-9 and PSS (AUC=0.85 and AUC=1, respectively), followed closely by DT (AUC=0.84 and AUC=1, respectively) and AdaBoost (AUC=0.82 and AUC=0.99, respectively). The RF model had the highest AUC to predict BAI (AUC=0.93), followed closely by AdaBoost (AUC=0.92). AdaBoost might be the algorithm of choice for the 3 outcomes, as it is fairing a close second best for BAI and a close third best for PHQ-9 and PSS. Gradient boosting and AdaBoost are both good choices to predict the intervention success for the 3 outcomes. It might be argued that AdaBoost might be preferable, given that it is usually less prone to overfitting than gradient boosting; however, there is no need to use the same algorithm to build the 3 predictors for the 3 outcomes.

We could not make a direct comparison with other studies that measured the 3 outcomes among university students using the same validated scales (PHQ-9, BAI, and SPSS). However, for PHQ-9, the performance of our model is higher than the one found in a previous study among adults in Korea using the Center for Epidemiologic Studies—Depression Scale 11 (AUC=0.87 and accuracy=0.86) [40] as well as the one found in a study in the United States that defined the success of the intervention as a 5-point reduction in PHQ-9 or a 4-point reduction in the General Anxiety Disorder screener-7 values (AUC=0.60 and accuracy=0.71) [35]. Regarding anxiety, the predictive model developed in this study had a higher performance (accuracy=0.92) than another study that used the Self-Rating Anxiety Scale, which did not report AUC but reported an accuracy of 0.84.

### Feature Importance

Exposure to mindfulness videos was the most important factor in predicting the intervention’s success. This study has demonstrated a link between the MVC intervention’s success and exposure to mindfulness videos. It also confirms the results of the previous MVC pilot study that proved that exposure to mindfulness videos alone, without interaction between participants via an online discussion forum and without weekly videoconferencing with a coach, effectively reduced symptoms of depression, anxiety, and stress [26]. In other words, it indicates the ability of MVC to be deployed at a large scale without an increase in human resources. Scalability is a critical factor for eHealth intervention deployment in large populations. This finding suggests that scaling up an effective e-mental health MVC is possible in a cost-effective manner; scalability is one of the recognized failures in eHealth implementations [42].

### Practical and Policy Implications

The MVC intervention does not provide clinical support; it is a platform that offers self-management of mental health symptoms (depression, anxiety, and stress). The MVC intervention proved to be effective [26-28] in reducing symptoms of depression, anxiety, and stress in university students. This study builds a predictive model that predicts intervention success using sociodemographic and self-reported measures; this will allow counseling services on university campuses to assess the usefulness of MVC for a particular student before taking the intervention and advise them accordingly to use MVC or to opt for another type of intervention. This will enable counseling services to personalize the advice to students’ profiles and allow students to manage their symptoms with the most appropriate intervention.

The other finding related to videos being the most important factor in predicting intervention success confirms the ability of MVC to be deployed at a large scale without an increase in human resources. The number of working hours is another important predictor of the success of the intervention. Although the provincial governments in Canada support university education, students must pay for their education and bear the cost of living. Not surprisingly, they work long hours, especially if they belong to a marginalized community. Our findings align with other studies that suggest that longer working hours outside the university and difficulty paying bills were recognized as predictors of poor mental health among students [43]. In Ontario, where the sample was taken, Statistics Canada recently reported an increased reliance of academic institutions on students’ fees in higher education to the extent that 54% of all college revenues in 2019/2020 were downloaded on students, which translates into an overall decline in public funding [44]. This situation pushed students to longer working hours; one can argue that since student debt has been recognized as negatively associated with mental well-being and academic outcomes [45,46], providing access to free higher education, supported by taxes such as in most of Europe, could enhance students’ mental well-being as it would relieve them from the need for long working hours.

### Strengths and Limitations

One of the strengths of this study is the ability to predict the intervention’s success based on a few demographics and one question about self-rated health. Hence, the predictive model can be used in real life to indicate the suitability of online mindfulness intervention for specific individuals and possibly suggest alternatives if the model predicts noneffectiveness. The excellent AUC and accuracy measures make the models suitable for implementation and evaluation in real-life scenarios. However, the ML models must be monitored continuously if implemented for daily use (eg, a counseling service) [47,48].

A limitation of this study is that it relied on research done on 1 site; future research with larger samples with participants from multiple universities and colleges would better test the generalizability of results as it allows us to test the effectiveness of the models on external data.

### Conclusions

Our results suggest that we can build high-performing models to predict MVC intervention effectiveness for depression, anxiety, and stress based on simple sociodemographics and self-reported features and that exposure to mindfulness videos is the most important predictor for the effectiveness of the intervention. Our findings provide evidence that scaling MVC can be done without additional cost for support and that the predictive models might be useful for professionals to advise students early enough on taking the intervention or choosing other alternatives.

## Abbreviations

AdaBoost: adaptive boosting
AUC: area under the curve
BAI: Beck Anxiety Inventory
DT: decision tree
KNN: k-nearest neighbor
LR: logistic regression
MCID: minimal clinically important difference
ML: machine learning
MVC: mindfulness virtual community
PHQ-9: Patient Health Questionnaire-9
PSS: Perceived Stress Scale
RCT: randomized controlled trial
RF: random forest
SVM: support vector machine
VC: virtual community

## Data Availability

The data sets generated and analyzed during this study are available from the corresponding author on reasonable request.
